# Correction: Novel polygenic risk score as a translational tool linking depression-related changes in the corticolimbic transcriptome with neural face processing and anhedonic symptoms

**DOI:** 10.1038/s41398-021-01277-y

**Published:** 2021-03-02

**Authors:** Klara Mareckova, Colin Hawco, Fernanda C. Dos Santos, Arin Bakht, Navona Calarco, Amy E. Miles, Aristotle N. Voineskos, Etienne Sibille, Ahmad R. Hariri, Yuliya S. Nikolova

**Affiliations:** 1 Campbell Family Mental Health Research Institute, Centre for Addiction and Mental Health (CAMH), Toronto, ON Canada; 2 Department of Psychiatry, University of Toronto, Toronto, ON Canada; 3 Department of Pharmacology and Toxicology, University of Toronto, Toronto, ON Canada; 4 Laboratory of NeuroGenetics, Department of Psychology and Neuroscience, Duke University, Durham, NC 27708 USA

**Keywords:** Diagnostic markers, Molecular neuroscience

Correction to: *Translational Psychiatry*

10.1038/s41398-020-01093-w published online 24 November 2020

The original version of this article unfortunately contained an error in the abstract. The correct sentence should be “We showed that T-PRS was associated with widespread reductions in neural response to neutral faces in women and increases in neural response to emotional faces and shapes in men (multivariate *p* < 0.01)”. The authors apologize for the mistake. The original article was revised.

