# Supplementary figures and images for: Novel polygenic risk score as a translational tool linking depression-related changes in the corticolimbic transcriptome with neural face processing and anhedonic symptoms
Source: Transl Psychiatry. 2020 Nov 24;10:410. doi: 10.1038/s41398-020-01093-w (PMC7686479; doi:10.1038/s41398-020-01093-w)

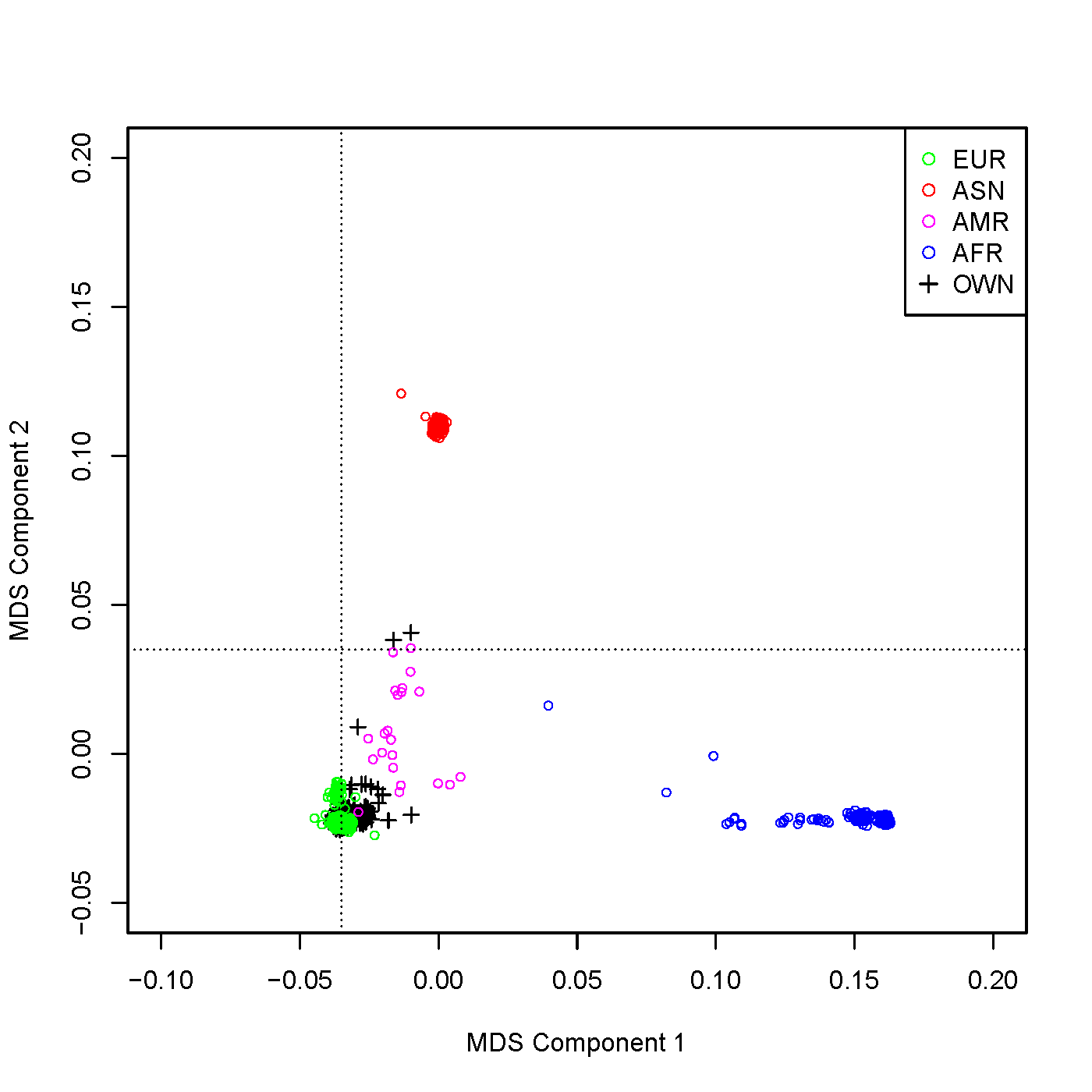

Supplement: Supplementary file 3 — Supplementary Figure 2 [file 41398_2020_1093_MOESM3_ESM.tif]

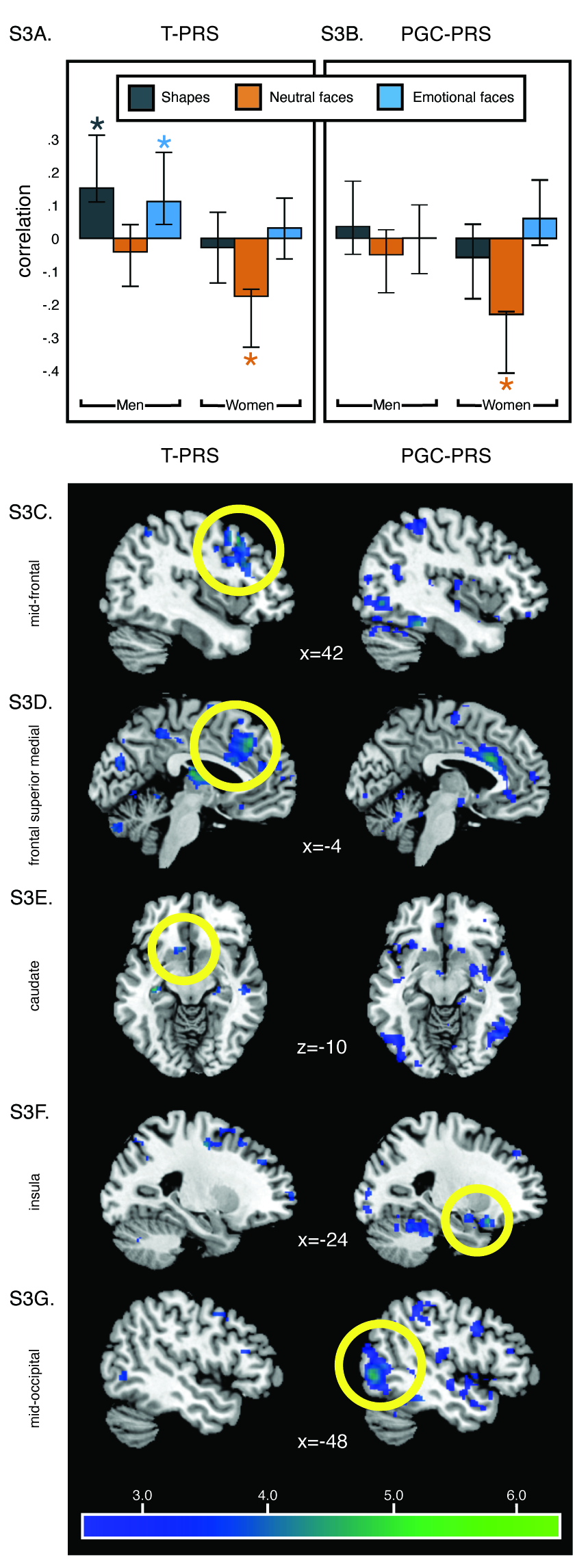

Supplement: Supplementary file 4 — Supplementary Figure 3 [file 41398_2020_1093_MOESM4_ESM.tif]

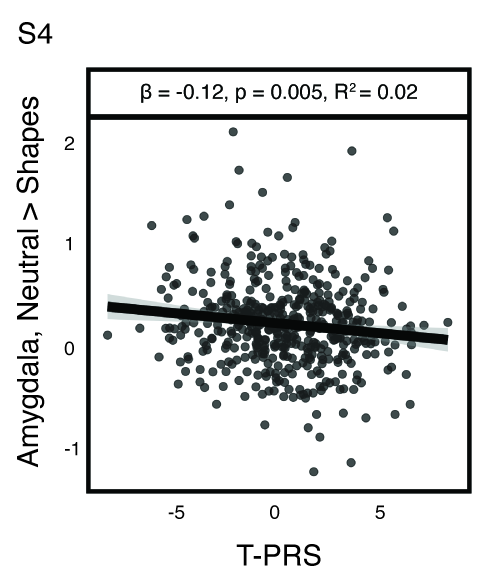

Supplement: Supplementary file 5 — Supplementary Figure 4 [file 41398_2020_1093_MOESM5_ESM.tif]

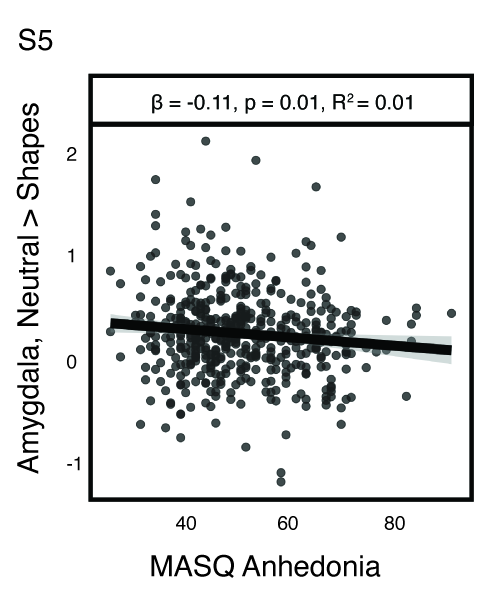

Supplement: Supplementary file 6 — Supplementary Figure 5 [file 41398_2020_1093_MOESM6_ESM.tif]

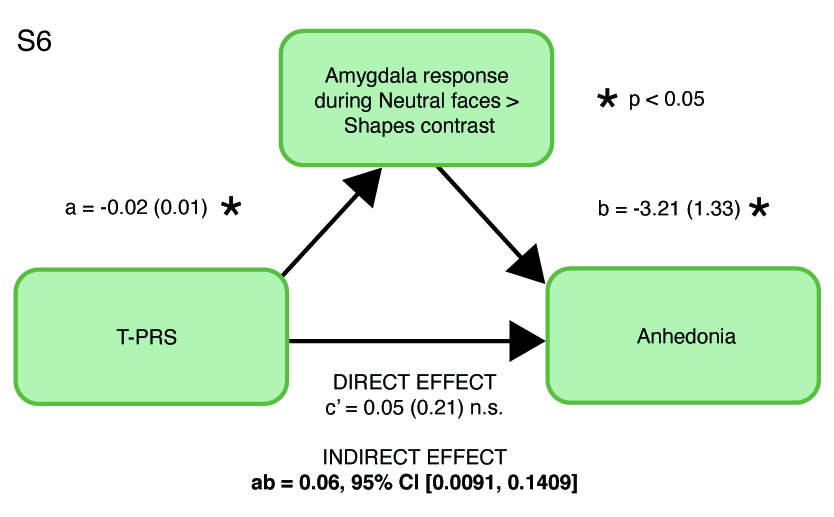

Supplement: Supplementary file 7 — Supplementary Figure 6 [file 41398_2020_1093_MOESM7_ESM.tif]
